# Stroke units, certification, and outcomes in German hospitals: a longitudinal study of patient-based 30-day mortality for 2006–2014

**DOI:** 10.1186/s12913-018-3664-y

**Published:** 2018-11-22

**Authors:** Christoph Pross, Elke Berger, Martin Siegel, Alexander Geissler, Reinhard Busse

**Affiliations:** 1 Department of Health Care Management, Berlin University of Technology, Administrative office H80, Str. des 17. Juni 135, 10623 Berlin, Germany; 2 Department of Empirical Health Economics, Berlin University of Technology, Berlin, Germany; 3 European Observatory on Health Systems and Policies, Brussels, Belgium

**Keywords:** Stroke, Stroke unit, Hospital specialization, Certificate, Accreditation, Stroke outcomes

## Abstract

**Background:**

Treatment of stroke patients in stroke units has increased and studies have shown improved outcomes. However, a large share of patients in Germany is still treated in hospitals without stroke unit. The effects of stroke unit service line, and total hospital quality certification on outcomes remain unclear.

**Methods:**

We employ annual hospital panel data for 1100–1300 German hospitals from 2006 to 2014, which includes structural data and 30-day standardized mortality. We estimate hospital- and time-fixed effects regressions with three main independent variables: (1) stroke unit care, (2) stroke unit certification, and (3) total hospital quality certification.

**Results:**

Our results confirm the trend of decreasing stroke mortality ratios, although to a much lesser degree than previous studies. Descriptive analysis illustrates better stroke outcomes for non-certified and certified stroke units and hospitals with total hospital quality certification. In a fixed effects model, having a stroke unit has a significant quality-enhancing effect, lowering stroke mortality by 5.6%, while there is no significant improvement effect for stroke unit certification or total hospital quality certification.

**Conclusions:**

Patients and health systems may benefit substantially from stroke unit treatment expansion as installing a stroke unit appears more meaningful than getting it certified or obtaining a total hospital quality certification. Health systems should thus prioritize investment in stroke unit infrastructure and centralize stroke care in stroke units. They should also prioritize patient-based 30-day mortality data as it allows a more realistic representation of mortality than admission-based data.

**Electronic supplementary material:**

The online version of this article (10.1186/s12913-018-3664-y) contains supplementary material, which is available to authorized users.

## Background

Stroke is the second-leading cause of death worldwide [1]. Recent data shows an incidence of about 16 million first-ever strokes annually, resulting in 5.7 million deaths, substantial long-term disabilities and significant long-term care costs [2]. Worldwide, the substantial stroke incidence, associated deaths and resulting medical and economic costs make it a truly global disease burden [3, 4].

The latest OECD Health Care Quality Indicator data suggest that admission-based mortality rates for ischemic stroke have decreased in the past decade, but stark cross-country outcome differences (6-fold variation) remain [5]. In Germany, an analysis of admission-based data showed an almost 20% reduction of raw and standardized mortality ratios (SMR) between 2005 and 2010. The study’s authors concluded a possible relation to improved primary and secondary prevention as well as increased treatment in specialized stroke units (SUs) [6]. The latter provide specialized acute and rehabilitation care with co-located and dedicated interdisciplinary teams of neurologists, internists, neuro- and vascular surgeons, and radiologists. 24/7 access to radiology (e.g. CT scanners) and thrombectomy equipment is also often included. SU care has been shown to improve both short- and long-term stroke outcomes [6–8], and reduce overall stroke treatment cost [9, 10].

In contrast, the evidence of a positive relationship between total hospital quality (THQ) certification and outcomes is mixed and incomplete. For stroke and acute myocardial infarction (AMI), deliveries, and hip fractures, a 2014 study found a positive association between certified THQ management systems and clinical leadership, systems for patient safety, and clinical review, but not for clinical practice [11]. Similarly, a study of the Joint Commission on Accreditation of Healthcare Organization (JCAHO) certification found risk-adjusted mortality rates improved in a cross-section analysis of 965 hospitals in 1996 and 1997 [12]. However, most studies find a weaker or non-existent effect between THQ and hospital outcomes [11, 13], and a more significant effect between service line quality systems and quality indicators (e.g. for stroke and AMI) [7, 13, 14].

Studies with a robust fixed effect framework, large hospital panel and patient-based outcome data - including for the period after hospital discharge - are rare. Further, while certification schemes continue to grow, the relationship between certification and hospital care outcomes remains inconclusive [15]. Studies have often examined the link between certification and process measures of care, but not the (or found only a weak) association between certification and outcome measures of care. To our knowledge, no study exists that differentiates outcomes for stroke care in a (i) conventional model, (ii) non-certified SU model, (iii) certified SU model, and (iv) hospitals with certified SU and/or additional THQ certification model, based on a large patient-based panel dataset.

To examine the influence of SU infrastructure and process specialization and certification on quality of stroke care, we rely on Donabedian’s structure, process, and outcome framework, in which outcomes are influenced by hospital structures and processes [16]. Stroke care is a particularly apt example to test this relationship since SU set-up and certification require substantial structural and process standards to be met. Therefore, we explore whether treatment of stroke in specialized facilities (i.e. SUs) improves quality and thereby warrants substantial investment at hospital and health system level. Likewise, we ask whether an additional SU certification further improves stroke care outcomes. We also examine if THQ certification and case volumes influence the relationship between SU specialization, certification, and stroke outcomes.

## Methods

### Data

We linked hospital data from different sources based on standardized institutional codes, which are unique mandatory identifiers for each hospital in Germany. First, we obtained structural hospital data (e.g. case volume, hospital teaching status, type of ownership) for the available years 2006, 2008, 2010, 2012, 2013, and 2014 from the German mandatory quality monitoring system, operated by the executive authority of the German health care system, the Federal Joint Committee (Gemeinsamer Bundesausschuss, G-BA). The G-BA provides publicly available hospital report cards for research purposes upon request via XML files on hospital and annual level.

Second, we integrated risk-adjusted, patient-based stroke outcome data (for the stroke diagnoses I. intracerebral hemorrhage, ICD Code I61; II. ischemic stroke, I63, and III. stroke not specified as hemorrhage or ischemic, I64) from the Quality Assurance with Routine Data (Qualitätssicherung mit Routinedaten, QSR) program. The QSR is operated by the AOK, the largest German sickness fund, and employs routine in- and outpatient data of AOK insured patients. It provides a risk-adjusted 30-day SMR, comparing observed vs. expected events. For risk-adjustment purposes, the QSR calculates 30-day expected mortality by means of logit regressions which includes patient-specific risk-factors like age, gender, and a set of comorbidities [17, 18]. To ensure comparability across years, we applied the 2014 logit risk-adjustment model to the AOK patient data for all data years.

Third, we included information on SU certification from the German Stroke Society (Deutsche Schlaganfall Gesellschaft, DSG), the premier German SU certification scheme [19]. The data provides information on which hospitals have DSG-certified SUs and the period of certification. A DSG certificate, granted for three years, requires minimum patient volume, minimum volume of certain interventions, staff level resources, and training obligations. Hospitals with non-certified SUs were identified by two specific procedure codes (OPS 8-891and 8-89b), which capture provision of complex stroke care [20].We assumed the existence of a SU when a hospital reported at least ten such procedures per year [6]. Structural standards are generally higher for DSG certification than for documenting complex stroke procedures.

Fourth, we integrated data from the THQ certificate Cooperation for Transparency and Quality in Health Care (Kooperation für Transparenz und Qualität im Gesundheitswesen, KTQ), comparable to JCAHO accreditation. Central components include continuous quality improvement in: patient orientation, employee orientation, patient safety, quality management, communication, transparency, and leadership [21]. Like the DSG SU certificate, the certificate is granted for 3 years. Hospital specific information on both certification schemes were provided from the mentioned organizations and integrated via standardized institutional codes and address information.

### Empirical strategy

Based on Donabedian’s quality framework [16], we hypothesize better stroke outcome quality for hospitals that organize care through: (ii) a dedicated SU facility, (iii) SU certification, and (iv) total hospital quality (THQ) certification, relative to the (i) conventional, non-SU care model. We employ a fixed effects model with a within-regression estimator at hospital level. To quantify the influence of (certified) SU care on stroke outcomes, we regress the log of stroke 30-day SMR (*SMR*_*it*_) on separate dummy variables, specifying the existence of a SU (*SU*_*it*_), a DSG-certified SU (*acc* _ *SU*_*it*_) and a THQ certification (*acc* _ *THQ*_*it*_). We add the log of stroke case volume (*stroke* _ *CV*_*it*_) to model stroke treatment experience, and a flattening learning curve. We include the share of stroke patients relative to all patients treated to account for relative importance of and organizational focus on stroke care. Hospital beds (*beds*_*it*_), dummy variables for hospital teaching status and ownership type, and a category medical specialization (CMS) [22] index reflect important time-variant characteristics. For time-variant trends that affect each hospital equally such as technological advances, regulatory changes, and judicial decisions, we specify time effects (*τ*_*t*_), excluding 2006 as the reference year. To adjust for the optimal level of stroke quality of care with a 0 SMR value (0 observed mortality), we adapt Battese’s (1997) approach to include an dummy explanatory variable (${D}_{it}^{SMR}$), which takes on the value of 1 when the SMR is 0, and add ${D}_{it}^{SMR}$ to *SMR*_*it*_ before taking the log [23]. We further adjust for the fact that hospitals treat variable amounts of stroke patients using AOK patient stroke case volume as analytical weights. The main model is specified in Eq. 1:

1 $$ \log \left({SMR}_{it}\right)={\beta}_0+{\beta}_1{D}_{it}^{SMR}+{\beta}_2{SU}_{it}+{\beta}_3{certSU}_{it}+{\beta}_4{certTHQ}_{it}+{\beta}_5\log \left({stroke}_{CVit}\right)+{\beta}_6{\frac{ stroke\ cases}{all\  cases}}_{it}+{\beta}_7{beds}_{it}+{\beta}_8{CMS}_{it}+{\beta}_9{teach}_{it}+{\beta}_{10}{private}_{it}+{\beta}_{11}{public}_{it}+{\alpha}_i+{\tau}_t+{\varepsilon}_{it} $$

In addition to the variables specified above, *β*_0_ is the intercept, *α*_*i*_ is individual time invariant hospital-fixed effects, and *ε*_*it*_ is the error term. To assess result robustness, we further estimate the model using the log of the number of SU complex procedures instead of the dummy indicator variable for stroke units. The data comprise repeated measurements at the hospital level which may involve autocorrelation in the error term *ε*_*it*_. A Hausman test indicates that a random effects specification would likely yield inconsistent estimates. We therefore use hospital fixed effects *α*_*i*_ to control for unobserved hospital characteristics and avoid inconsistencies. Testing the time-fixed effects *τ*_*t*_ for joint significance indicates systematic differences in mortality across years. All statistical inferences are based on heteroscedasticity- and autocorrelation-consistent estimates for the standard errors.

## Results

Between 2006 and 2014 our sample includes on average 1243 hospitals per year (Table 1). Because of hospital closures and mergers the number of hospitals within our sample decreased by 13% from 1331 in 2006 to 1162 in 2014, 726 stroke-treating hospitals had no SU, 436 hospitals did, of which 222 SUs were DSG-certified, and 280 hospitals were THQ-certified. On average, hospitals treat 227 stroke patients per annum and have a 30-day stroke SMR of 0.99, a reduction of approximately 13% since 2006. In 2014, our hospital sample includes 86% of all hospitals that recorded at least 2 stroke diagnoses. The discrepancy (Table 1) is due to QSR data availability and the G-BA’s 2010 shift to reporting at site level, resulting in increases to the number of hospitals and sites in the overall, non-QSR sample.

**Table 1:** Overview main variables over time from 2006 to 2014

	2006	2008	2010	2012	2013	2014
Number of hospital observations^a^	1331	1292	1244	1228	1203	1162
Average 30-day stroke SMR^b^	1.12	1.09	1.06	1.05	1.02	0.99
Number of hospitals with SU^c,d^	276	337	439	414	423	436
Number of ospitals with certified SU^d,e^	162	189	177	211	220	222
Number of hospitals with THQ certification^d^	383	434	398	360	334	280
Average stroke case volume^f^ per hospital	167	188	202	215	219	227
Share stroke cases/inpatient cases (in %)	3.2	3.3	3.4	3.5	3.4	3.6
Numb. of hospitals with teaching status (%)	511 (38)	557 (43)	581 (47)	619 (50)	625 (52)	642 (55)
Average number of hospital beds	331	334	341	347	348	355
Average CMS specialization index^f^	1.35	1.34	1.46	1.38	1.37	1.40
Number of orivate, for-profit hospitals (%)	225 (17)	237 (18)	245 (20)	257 (21)	255 (21)	255 (22)
Number of private, non-profit hospitals (%)	592 (44)	574 (44)	551 (44)	527 (43)	514 (43)	477 (41)
Number of public hospitals (%)	514 (39)	481 (37)	448 (36)	444 (36)	434 (36)	430 (37)
Number of hospitals with stroke diagnoses^g^	1332	1362	1329	1,37	1341	1344

^a^all observations that have QSR SMR stroke outcome data

^b^weighted by the AOK stroke patient volume for each hospital

^c^based on more than 10 documented complex stroke procedures (OPS codes 8_891 und 8_89b), for 2014 461 SU exist in full sample independent on whether QSR data is available

^d^and have QSR SMR stroke outcome data (especially for THQ overall more certified hospitals in Germany)

^e^DSG SU certification suspended in 2008 and part of 2009, which led to a backlog of (re-) certification applications and a reduction in DSG certified hospitals in data year 2010

^f^based on ICD stroke diagnoses I61 (hemorrhage), I63 (ischemic) and I64 (not further specified)

^g^All hospitals which have coded 2 or more stroke ICD cases. Discrepancy in number of observations due to QSR data availability and G-BA reporting for multiple sites starting in 2010 and becoming mandatory in 2012 and 2013

**Fig. 1:**
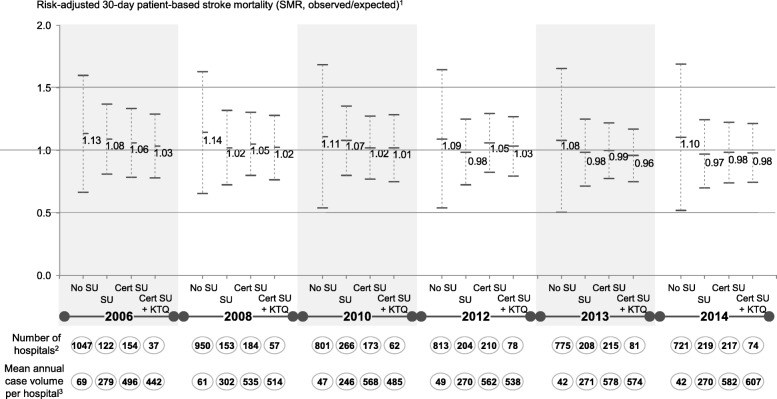
Median and standard deviation (above and below median) for the 30-day stroke SMR and hospitals with a conventional care model (‘No SU‘), a SU facility (‘SU’), a certified SU (‘Cert SU’) and a certified SU within a hospital with a KTQ THQ certificate (‘Cert SU + KTQ’). Note: 1. QSR stroke volume applied as analytical weights; 2. Number of hospitals and associated hospital sites; 3. Mean annual stroke ICD case volume including diagnoses I61 (hemorrhage), I63 (ischemic) and I64 (not further specified)

Figure 1 presents the weighted median and standard deviation (SD) of the SMR for the respective hospital sub-groups with conventional stroke care (‘No SU’), a dedicated SU care model (‘SU’), a certified SU (‘Cert SU’) and a certified SU within a hospital with a KTQ THQ certificate (‘Cert SU + KTQ’).

Hospitals that treat stroke patients in a conventional model have the highest SMR and the largest outcome variation (i.e. SD). Their number reduces from 1047 hospitals in 2006 to 721 in 2014 and their average stroke patient volume declines from 69 to 42 patients; however, in 2014 30,000 stroke patients are still treated at hospitals with a subpar care model and a substantially higher risk of death.

Compared to the conventional mode, the outcome quality improves for patients treated in a stroke unit. Both the median SMR and the outcome variation are substantially reduced. Over time, the median SMR for all subgroups improves, however outcome variation remains roughly constant.

In 2006 and 2008, the SMR is lower in both certified SU care models relative to the non-certified SU. However, from 2010 to 2012, the median SMR for hospitals with a non-certified SU decreased from 1.07 to 0.98, while for hospitals with a certified SU or both SU and THQ certifications it increased to 1.05 and 1.03. More than 30 larger hospitals with a relatively high 30-day SMR received a SU certification between 2010 and 2012 and decreased their 30-day SMR, which lowered the overall average in the following years, but pushed up the SMRs for the certification subgroups.

Table 2 presents descriptive statistics for the relevant empirical model variables summing across all years.

**Table 2:** Descriptive statistics, all years (Mean, standard deviation, minimum, maximum)

	Mean	SD	Min	Max
Number of observations^a^ (2006–2014)	7462			
Average stroke 30-day SMR^b^	1.06	0.4	0.0	17.8
Log average stroke 30-day SMR^b^	0.02	0.3	−2.1	2.9
Hospitals with a specialized SU	0.31	0.5	0.0	1.0
Average complex stroke procedures	144.96	303.0	0.0	6443.0
Log average complex stroke procedures^b^	−11.83	12.3	−20.7	8.8
Hospitals with DSG-certified SU	0.16	0.4	0.0	1.0
Hospitals with KTQ-TQH certification	0.29	0.5	0.0	1.0
Average case volume stroke cases	202.17	279.0	0.0	5327.0
Log average case volume stroke cases^b^	3.84	4.1	−20.7	8.6
Share stroke cases / inpatient cases	0.03	0.1	0.0	0.8
Hospitals with teaching status	0.47	0.5	0.0	1.0
Average number of hospital beds	342.23	302.0	0.0	3213.0
CMS specialization index	1.35	0.8	0.0	4.3
Private, for-profit hospitals	0.20	0.4	0.0	1.0
Private, non-for-profit hospitals	0.43	0.5	0.0	1.0
Public hospitals	0.37	0.5	0.0	1.0

^a^all hospitals that have QSR SMR stroke outcome data

^b^QSR stroke patient volume applied as analytical weights; 0.000000001 or 1 added before taking the log to avoid losing observations with 0 values, results are similar for both

Table 3 presents regression results of our main model (M1). SU care is associated with a 5.6% lower 30-day SMR, while SU or THQ certification shows no significant additional effect on stroke outcomes. Neither stroke volume nor the share of stroke cases relative to all inpatient cases has a significant effect on SMR. The time fixed effects for years 2013 and 2014 have negative and significant coefficients (− 0.05***, − 0.08***). We consider M1 our main model as it implements our empirical strategy and has the lowest Bayesian Information Criterion (BIC) [24].

**Table 3:** Regression results main model M1 (beta, lower and upper confidence interval)

	M1
Log dummy	−0.059* (−0.112, −0.007)
Stroke unit (SU)	− 0.056** (− 0.092, − 0.021)
SU certification	− 0.005 (− 0.036, − 0.026)
THQ certification	0.016 (− 0.019, 0.051)
Log stroke case volume	− 0.003 (− 0.010, 0.003)
Share stroke ICD/all ICD	− 0.054 (− 0.743, 0.635)
Hospital beds	− 0.000 (− 0.000, 0.000)
Teaching hospital status	0.007 (− 0.041, 0.056)
CMS ICD specialization index	− 0.003 (− 0.039, 0.033)
Private, for-profit hospitals^a^	− 0.022 (− 0.144, 0.101)
Public hospitals^a^	− 0.011 (− 0.138, 0.117)
Log all OPS stroke procedures	–
Interaction SU and THQ certification	–
Constant	0.182* (0.030, 0.333)
R2-within	0.016
R2-between	0.032
R2-overalll	0.018
BIC	1084
Intraclass correlation	0.512
F-statistic	3.9
Number of observations	7376

QSR stroke patient volume applied as analytical weights; *p < 0.05, **p < 0.01, ***p < 0.001; time-fixed effects not displayed separately (in M1, β_2008_ = − 0.02, β_2010_ = − 0.03, β_2012_ = − 0.03, β_2013_ = − 0.05***, β_2014_ = − 0.08***), test for joint significance of time effects in M1 with F-statistic of 5.59

^a^private (non-profit) hospitals serve as reference category

For model robustness, we ensure consistency of our results when using alternative variable, sample, and model specifications M2 to M9 (see Additional file 1).

## Discussion and limitations

### Discussion

Our analysis confirms the positive trend over time of SMR reduction after stroke in Germany, although to a much lower degree than prior studies have shown [6]. This can be attributed to the use of patient-based 30-day mortality data, including time after patient discharge. This data enables a cross-sectoral perspective on stroke care and demonstrates the shortcomings of admission-based data.

The descriptive stroke SMR trends for the different hospital sub-groups suggest progressively better stroke outcomes in hospitals with SU infrastructure, a SU that is also DSG-certified, and a certified SU within a THQ certified hospital. Results of the fixed effects regression models also show that having a SU alone significantly enhances outcome quality of care. The results align with previous research and confirm the benefits of treating patients in a dedicated SU facility [7, 8, 14].

Conversely, both certifications do not show significant effects. The structural and process differences between non-certified and certified SUs might be too small to show significant impact, and the overall hospital quality management improvements associated with the THQ certification might not be meaningful enough to influence outcomes in emergency medical conditions such as stroke.

On a health system level, our results question why a large share of German stroke patients is still treated in non-specialized facilities, and, related, why the shift towards a centralized stroke treatment model is sluggish [6]. Our findings suggest that treating all stroke patients at hospitals with a SU may result in a decrease in the absolute 30-day stroke mortality by 5.6%, from 16.2 to 15.3% even after adjusting for case volume and share of stroke cases. For those roughly 50,300 stroke patients currently treated at hospitals without SUs, this would correspond to 460 fewer annual stroke-related deaths. Considerable reductions in stroke-related disabilities and in medical and economic costs are additional expected benefits [7].

Experience in other European countries demonstrates the positive outcome impact of stroke care centralization in SUs [25, 26]. Underpinning the centralization argument is the positive volume-outcome relationship, which has also been shown to hold for stroke [27]. In the mid-term, national and regional policy makers should ensure that all stroke patients are treated in SUs by requiring SU infrastructure for stroke care and centralizing stroke care with hospitals that already operate a well-performing SU.

The German certification of SUs sets high procedural, personnel, and infrastructural standards; however, as above, in contrast to expectations, the SU service line certification shows no additional significant improvement with 30-day stroke SMR when non-certified SU existence is controlled for. Several explanations are possible. First, DSG certification confirms the SU set-up externally, with some additional staffing and process requirements. These enhancements might not have a large enough additional effect on the 30-day mortality compared to the standard SU characteristics.

Second, mortality is a valid and well-accepted outcome parameter [28], but it is only one of the outcomes that matters in stroke care [29]. Others, such as readmissions, degree of disabilities, and quality of life are also important [7, 29]. Standardized and risk-adjusted data for these outcome parameters are not currently available in Germany. Certified SUs, however, might have better outcomes for these indicators because the DSG certification takes a holistic approach, focusing on reducing disabilities after stroke [19]. Third, certified SU might have improved outcomes over a longer timeframe than the 30 days after hospital admission examined here.

Likewise, certified SUs might provide care for more severe patients, as they have on average substantially higher case volumes (Fig. 1). While the standardized 30-day stroke mortality is adjusted for co-morbidities, stroke severity (e.g. National Institutes of Health Stroke Scale from 0 to 42) is not fully reflected by administrative data [30]. However, the impact of severity adjustment on risk-adjusted indicators that already are adjusted for co-morbidities, age and other patient characteristics has been shown to be limited [31]. Lastly, the suspension of the DSG SU certification process in 2008 and first months of 2009, which resulted in delays for about 100 re- or new stroke unit certifications [32], might have also reduced the effectiveness of the DSG certification for the time span 2008–2012 and the amount of 30-day stroke SMR improvement attributable to the DSG certification.

THQ certification showed no additional significant effect on 30-day stroke mortality, in line with previous studies in other countries [11, 13]. The primary purpose of this certification is the general improvement of hospital quality management; its achievement might not be appropriately reflected by 30-day mortality in one specific emergency condition. Other measures such as patient safety, patient and employee responsiveness and satisfaction, and operational efficiency at the hospital level might be more affected by THQ certification. For example, Lindlbauer et al. (2016) show improved technical efficiency for THQ-certified hospitals. A downward bias of the THQ effect could be possible due to the fact that no consolidated and standardized data on ISO 9001 certification, which is a universal quality certificate also applied in hospitals, is available. Hospitals without a KTQ certification might alternatively have an ISO 9001 THQ certification even though they appear without THQ certification in our dataset. However, the number of ISO 9001 certifications is likely substantially smaller compared to the KTQ-certified hospitals [22].

Lastly, there are benefits from certification schemes that are not captured by outcome data. Both the SU and the THQ certification provide quality signals for patients, emergency teams, and admitting physicians, which can facilitate hospital choice decisions.

### Limitations of this study

Besides the limitations mentioned above, the results of this study should be viewed considering some data and methodological limitations. The validity of self-reported hospital data might be compromised, due to reputational concerns by hospitals and different coding practices. Annual, random validity checks and cross-checks with administrative patient data, demonstrated for 5% of hospital reports some validity issues affecting 15–60% of the examined reporting data(26, 57).

The analyzed post-discharge timeframe of 30 days for stroke mortality provides substantial information on outcome quality, but an extended period like365 days might provide additional insights. While the AOK QSR indicators have some advantages, they only rely on data for patients insured by the AOK sickness fund. This might lead to biased outcome indicators, but the high share of AOK insured patients in all German hospitals (35% average market share) and results from previous studies (58) demonstrate the representativeness of the AOK QSR data.

Even though the outcome data is risk-adjusted for a large set of comorbidities and age, some bias might be affecting the results as the outcome data is not fully adjusted for severity. This might especially affect certified stroke unit hospitals as they could receive more severe cases, also via transfer from non-certified stroke units, leading to higher mortality that is not accounted for in the patient-based risk adjustment. Therefore, the effect of a SU certification or a full hospital certification is possibly underestimated in our data.

## Conclusions

Our results substantiate the positive effect of SU treatment on stroke outcomes, based on a fixed effects model and large multi-year hospital sample, suggesting that hospital and health system investment in SUs improve stroke outcomes. SUs may help save numerous life-years, reduce stroke associated disabilities, and lower long-term stroke treatment cost considerably. Germany can learn from other country examples regarding centralization and (mandatory) emergency protocols for stroke treatment. As the first study to distinguish the potential effects of SU existence, SU certification and THQ certification, we do not find a significant effect for SU certification or THQ certification on top of the large and significant effect for SU specialization.

Our research contributes to the literature on outcomes and operational research and how hospital quality of care can be improved through structural and process enhancements. The results have implications for the organization of stroke care in other countries as well as the academic and professional debate around the benefits of infrastructure specialization and certification in health care. Additional research can examine the effect of specialization and service line certification on other stroke outcome measures (e.g. disabilities) and outcomes in other treatment areas, such as cardiology or oncology specialized treatment units. Likewise, the effect of THQ can also be examined with other outcome indicators, with additional information on other THQ certifications and for other more elective treatment areas, where a THQ certification might possibly show a higher impact.

## Additional file

Additional file 1: Material S1. Robustness checks. The supplementary material S1 includes results of model specifications (M2 to M9) as robustness checks. (DOCX 40 kb)

## Abbreviations

AMI: Acute myocardial infarction
BIC: Bayesian Information Criterion
CMS: Category medical specialization
DSG: Deutsche Schlaganfall Gesellschaft (German Stroke Society)
G-BA: Gemeinsamer Bundesausschuss (Federal Joint Committee)
JCAHO: Joint Commission on Accreditation of Healthcare Organization
KTQ: Kooperation für Transparenz und Qualität im Gesundheitswesen (Cooperation for Transparency and Quality in Health Care)
QSR: Qualitätssicherung mit Routinedaten (Quality Assurance with Routine Data)
SD: Standard deviation
SMR: Standardized mortality ratios
SU: Stroke unit
TQM: Total hospital quality management
WIdO: Wissenschaftliches Institut der AOK (Research Institute of the AOK SHI fund)
