# Supplementary material for: Stroke units, certification, and outcomes in German hospitals: a longitudinal study of patient-based 30-day mortality for 2006–2014
Source: BMC Health Serv Res. 2018 Nov 22;18:880. doi: 10.1186/s12913-018-3664-y (PMC6249823; doi:10.1186/s12913-018-3664-y)
Supplement: Supplementary file 1 — Material S1. Robustness checks. The supplementary material S1 includes results of model specifications (M2 to M9) as robustness checks. (DOCX 40 kb) [file 12913_2018_3664_MOESM1_ESM.docx]

**Supplementary Material S1: Robustness checks**

To ensure robustness, we test the consistency of our results when using alternative variable, sample, and model specifications. Results of the robustness tests are displayed in Table S1**.** In M2, we replace the SU dummy with the log of complex stroke procedures. The effect of the number of complex SU procedures is significant at the 10% level and has a quality-enhancing negative effect on standardized 30-day stroke mortality (-0.002**). The BIC increases slightly for model M2. In M3, we add the interaction effect for service line and THQ certification, which is also insignificant and slightly increases the effect size of the SU certification dummy variable.

The effects are generally consistent across model specifications, both in size and significance. The models display low R-squared measures, however, since fixed effects regressions washout the explanatory effects of the intercepts, they generally show a much lower R-squared compared to ordinary least squares models. When limiting the sample to observations with more than 10 or 20 QSR stroke cases per year (M4 and M5), the effect magnitude is reduced. Increasing the number of QSR cases required for model inclusion eliminates many hospitals with very small case volumes, which can reduce the impact of statistical chance on the SMR [26]. But removing 1,800 and 2,882 of hospital observations from the sample also reduces statistical power and excludes many of the hospitals with little experience in treating stroke patients. We further alter the specification of our main SU dummy variable to ensure our results hold with different SU thresholds. Halving and doubling the complex stroke procedure threshold (M6 and M7) for the SU dummy specification does not change the results noteworthy.

We also test the effect of the two certification schemes without the SU infrastructure measure (M8). Even without the SU variable, both the SU service line certification and the THQ certification remain insignificant. Furthermore, to independently check the effect of THQ certification on AMI as another emergency condition, we estimate equation 1 without the SU infrastructure and certification dummy variables and replace the relevant stroke variables with the corresponding AMI variables (M9). As before, the coefficient for the THQ certification dummy remains insignificant. Furthermore, when the 30-day SMR is substituted by the simple 30-day mortality rate as the dependent variable, the results remain comparable. Likewise, when the main model (M1) is run without AOK patient case volume as analytical weights, then the effect size doubles to -10.8% (-0.108***). When replacing the AOK stroke patient number as analytical weight with the overall stroke ICD patient number, the results also remain comparable with M1.

*Insert Table S1 about here*

| **Table S1:** Regression results for robustness models M2-M9 | | |  |  |  |  |  |  |
| --- | --- | --- | --- | --- | --- | --- | --- | --- |
|  | **M2** | **M3** | **M4** | **M5** | **M6** | **M7** | **M8** | **M9** |
| Log dummy | -0.06 (-0.11, -0.01) | -0.06 (-0.11, -0.01) | 0.18 (0.10, 0.26) | 0.26 (0.08, 0.44) | -0.06 (-0.11, -0.01) | -0.06 (-0.11, -0.01) | -0.05 (-0.11, -0.00) |  |
| SU facility (> 10 QSR pat. cases) |  | -0.06 (-0.09, -0.02) | -0.04 (-0.08, -0.01) |  |  |  |  |  |
| SU facility (> 20 QSR pat. cases) |  |  |  | -0.04 (-0.08, -0.01) |  |  |  |  |
| SU facility (>5 comp. stroke proc) |  |  |  |  | -0.06 (-0.09, -0.02) |  |  |  |
| SU facility (>20 com. stroke proc) |  |  |  |  |  | -0.06 (-0.09, -0.02) |  |  |
| SU certification | -0.01 (-0.04, 0.03) | -0.02 (-0.05, 0.01) | -0.00 (-0.03, 0.03) | -0.00 (-0.03, 0.03) | -0.01 (-0.04, 0.03) | -0.01 (-0.04, 0.03) | -0.01 (-0.04, 0.02) |  |
| THQ certification | 0.02 (-0.02, 0.05) | -0.00 (-0.04, 0.03) | 0.02 (-0.02, 0.06) | 0.02 (-0.02, 0.06) | 0.02 (-0.02, 0.05) | 0.02 (-0.02, 0.05) | 0.02 (-0.02, 0.05) | -0.02 (-0.06, 0.02) |
| Log (stroke case volume) | -0.00 (-0.01, 0.00) | -0.00 (-0.01, 0.00) | -0.00 (-0.01, 0.01) | -0.00 (-0.01, 0.01) | -0.00 (-0.01, 0.00) | -0.00 (-0.01, 0.00) | -0.00 (-0.01, 0.00) |  |
| Share stroke ICD/all ICD | -0.06 (-0.75, 0.63) | -0.07 (-0.76, 0.62) | 0.11 (-0.64, 0.85) | 0.36 (-0.35, 1.07) | -0.06 (-0.75, 0.63) | -0.05 (-0.73, 0.64 ) | -0.10 (-0.80, 0.60) |  |
| Log (hospital beds) | -0.00 (-0.00, 0.00) | -0.00 (-0.00, 0.00) | -0.00 (-0.00, 0.00) | -0.00 (-0.00, 0.00) | -0.00 (-0.00, 0.00) | -0.00 (-0.00, 0.00) | -0.00 (-0.00, -0.00) | -0.00 (-0.00, 0.00) |
| Teaching hospital status | 0.01 (-0.04, 0.06) | 0.01 (-0.04, 0.06) | 0.01 (-0.04, 0.06) | 0.00 (-0.05, 0.05) | 0.01 (-0.04, 0.06) | 0.01 (-0.04, 0.06) | 0.00 (-0.05, 0.05) | 0.02 (-0.04, 0.08) |
| CMS ICD | -0.00 (-0.04, 0.03) | -0.00 (-0.04, 0.03) | -0.01 (-0.040, 0.03) | -0.01 (-0.04, 0.03) | -0.00 (-0.04, 0.03) | -0.00 (-0.04, 0.33) | -0.00 (-0.04, 0.03) | 0.01 (-0.03, 0.06) |
| Private (for-profit) hospital^1^ | -0.02 (-0.14, 0.10) | -0.02 (-0.14, 0.10) | -0.03 (-0.16, 0.10) | -0.05 (-0.20, 0.09) | -0.02 (-0.14, 0.10) | -0.02 (-0.14, 0.10) | -0.03 (-0.15, 0.10) | 0.09 (-0.07, 0.25) |
| Public hospital^1^ | -0.01 (-0.14, 0.12) | -0.01 (-0.14, 0.11) | -0.02 (-0.15, 0.12) | -0.04 (-0.18, 0.11) | -0.01 (-0.14, 0.12) | -0.01 (-0.14, 0.12) | -0.10 (-0.14, 0.12) | 0.10 (-0.19, 0.38) |
| Log all OPS stroke procedures | -0.00 (-0.00, -0.00) |  |  |  |  |  |  |  |
| Interaction SU and THQ certify, |  | 0.06 (-0.01, 0.12) |  |  |  |  |  |  |
| Log dummy AMI |  |  |  |  |  |  |  | 0.00 (.) |
| Log (AMI case volume) |  |  |  |  |  |  |  | -0.02 (-0.07, 0.04) |
| Share AMI ICD/all ICD |  |  |  |  |  |  |  |  |
| Constant | 0.14 (-0.02, 0.29) | 0.19 (0.04, 0.34) | 0.16 (0.00, 0.32) | 0.17 (-0.01, 0.34) | 0.18 (0.03, 0.33) | 0.18 (0.03, 0.33) | 0.16 (0.01, 0.31) | 0.09 (-0.19, 0.38) |
| R2-within | 0.02 | 0.02 | 0.02 | 0.02 | 0.02 | 0.02 | 0.01 | 0.03 |
| R2-between | 0.03 | 0.03 | 0.00 | 0.01 | 0.03 | 0.03 | 0.02 | 0.03 |
| R2-overalll | 0.02 | 0.02 | 0.00 | 0.00 | 0.02 | 0.02 | 0.01 | 0.01 |
| BIC | 1088 | 1086 | 432 | -41 | 1084 | 1084 | 1090 | 2176 |
| Intraclass correlation | 0.51 | 0.51 | 0.55 | 0.62 | 0.51 | 0.51 | 0.51 | 0.62 |
| F-statistic | 3.8 | 4.0 | 5.0 | 4.0 | 3.9 | 3.9 | 3.4 | 7.0 |
| Number of observations | 7376 | 7376 | 5576 | 4494 | 7376 | 7376 | 7376 | 6188 |
| **Note**: QSR stroke patient volume applied as analytical weights; 1: private (non-profit) hospitals serve as reference category | | | | | | | | |
